# Health status among black African-born women in Kansas City: a preliminary assessment

**DOI:** 10.1186/s13104-015-1469-1

**Published:** 2015-10-05

**Authors:** Florence M. Ndikum-Moffor, Babalola Faseru, Melissa K. Filippi, Hou Wei, Kimberly K. Engelman

**Affiliations:** Department of Preventive Medicine and Public Health, University of Kansas Cancer Center, Center for American Indian Community Health, University of Kansas Medical Center, 3901 Rainbow Boulevard, MS 1056, Kansas City, KS 66160 USA; Department of Preventive Medicine and Public Health, Center for American Indian Community Health, University of Kansas Medical Center, 3901 Rainbow Boulevard, Kansas City, KS 66160 USA; Department of Family Medicine, Center for American Indian Community Health, University of Kansas Medical Center, 3901 Rainbow Boulevard, Kansas City, KS 66160 USA

**Keywords:** Women’s health, Health disparities, African-born women’s health

## Abstract

**Background:**

Health information and statistics for Black foreign-born women in the United States are under-reported or not available. Black foreign-born women typically are classified under the general category of African American, ignoring the heterogeneity that exists in the United States Black population. It is important to identify health issues and behaviors of African-born women to effectively address health disparities.

**Methods:**

Black African-born women (N = 29), 20 years or older completed a survey about general and women’s health, health history, acculturation, lifestyle, social and health challenges, beliefs about breast cancer. Data were analyzed using SPSS 14.0 software. Categorical variables were summarized with frequencies and percentages and continuous variables were summarized with means and standard variation. A Likert scale (strongly agree, agree, disagree, and strongly disagree) was used to assess beliefs about breast cancer.

**Results:**

Most (71.4 %) participants had a high school education or more, 70 % were employed, and 50 % had health insurance. Two-thirds received health care from primary care doctors, 20.7 % from health departments, and 39.3 % got annual checkups. Lack of jobs, healthcare cost, language barrier, discrimination, and child care were the top social issues faced by participants. High blood pressure, obesity, oral health, HIV/AIDS, and diabetes were indicated as the most common health problems. The percent of participants (60 %) that had not had a mammogram within the previous 2 years was more than the state average (24 %) for women 40 years and older reported by the Kansas Department of Health and Environment. The percent of participants (40 %) that had a mammogram within the previous 2 years was lower than the national average (73.2 %) for African American women.

**Conclusions:**

Study provides a snapshot of social concerns and health issues in an African population residing in Midwestern United States. Understanding the socio-cultural characteristics of this population is necessary to address health disparities.

## Background

### African-born women in the United States

The African immigrant/refugee population in the United States doubled between 2000 and 2010, with the number of women growing from 396,510 to 761,677 [1–3], and women making up about 50 % of the African immigrant/refugee population [4]. While the health status for Black women is reported in general [5], health information and statistics for Black foreign-born women are under-reported or not available [6], and are not distinguished from Black United States-born women.

Thus, there is a paucity of information about the health issues in this population of Black women. Black women in the United States continue to have the highest rates of breast cancer deaths. Various reasons for this increased mortality have been suggested including low screening rates, lack of awareness, and inadequate treatment [7]. However, data that describe breast cancer in black women in the US lump together US-born and foreign–born black women, undermining the differences in socio-cultural and psychosocial characteristics that may influence their perceptions and attitudes towards breast cancer diagnosis and utilization of screening services. Black African-born women have socio-economic and cultural beliefs that could differentially affect their lifestyle and health care access compared to Black United States-born women [6]. Additionally, some immigration laws make it more difficult for immigrants to gain access to health care compared to native United States citizens; for example, non-citizens who are legal residents are not Medicaid eligible until after 5 years as legal residents [8]. Recording and reporting health statistics data only by (black) race masks differences within the Black population and provides inadequate information to develop effective programs [9]. To remedy this conundrum, the United States Department of Health and Human Services created an African Data Work Group to collect, organize, share, and identify gaps in knowledge and information on African immigrants and refugees [10]. The objectives of this study were to gather information about health issues and health care access among African immigrant and refugee women, and assess women’s knowledge of and awareness about breast cancer.

## Methods

Black African-born women (N = 29) originally from countries in West, Central and East Africa, and living in the Kansas City (KC) metropolitan area completed a survey about general health, women’s health, oral health, diet and exercise, acculturation, health history, and beliefs about breast cancer. Participants were recruited during an educational event to increase breast and cervical cancer awareness among immigrant and refugee women from Africa. The event was advertised through social media, and by posting flyers at local African-churches, grocery stores, hair salons, and community organizations. The event was also advertised by word of mouth. While questions about health status and health care access were addressed to all participants, questions about breast cancer screening including mammography were addressed to women aged 35 and older. Informed consent to participate in the study was obtained from participants verbally before they completed the survey. The Institutional Review Board of the University of Kansas Medical Center approved the study. Data were analyzed using SPSS 14.0 software. Categorical variables were summarized with frequencies and percentages and continuous variables were summarized with means and standard variation. A four-level Likert scale (strongly agree, agree, disagree, and strongly disagree) was used to assess beliefs about breast cancer.

## Results and discussion

Participants were 20–68 years old (mean age = 39 years), had lived in the United States from 1 to 17 years; 29 % had lived in the United States for just 1 year and 29 % had lived in the United States for over 10 years. Most (70 %) were employed and 71.4 % had more than a high school education. Over half (55.6 %) of the participants indicated they always spoke a language other than English outside their homes. Two-thirds were married or living with a partner, and 78.6 % had at least one child (Table 1).

**Table 1:** Participant demographics (N = 29)

Characteristic	**N**	Percent
*Age* (in years, mean, SD)	*23*	(39.0 ± 11.4; mean age)
*Country of origin*	*26*	
Cameroon	10	38.5
Kenya	12	46.1
Nigeria	4	15.4
*Years in the US*	*28*	
1 year	8	28.6
2–4 years	5	17.8
5 years	3	10.7
6–10 years	4	14.3
Over 10 years	8	28.6
*Current living situation*	*28*	
Married/living with a partner	19	67.9
Divorced/separated/widowed	5	17.9
Never married	2	7.1
Other	2	7.1
*Highest educational attainment*	*27*	
Elementary/grade school	2	7.4
High school graduate/GED	4	14.8
Some college	1	3.7
2-year college graduate (AA degree)	9	33.3
4-year college graduate (BA/BS degree)	8	29.6
Graduate degree	3	11.1
*Employment status*	*27*	
Employed	19	70.4
Self-employed	2	7.4
Out of work for <1 year	1	3.7
Stay home mom	2	7.4
Unable to work	3	11.1
*Children*	*28*	
Yes	22	78.6

Italicized texts represent total number of respondents per characteristic

### Health care access

Access to health care, cancer screening and dental services by participants are summarized in Table 2. Noteworthy is that only 34.6 % of participants had private health insurance and 15.4 % had other types of insurance coverage, 28.6 % had never been to a dentist, and only 3 of 10 women (40 and older) were up-to-date on their mammograms. The percent of participants (60 %) that had not had a mammogram within the previous 2 years was more than the state average (24 %) for women 40 years and older reported by the Kansas Department of Health and Environment. The percent of participants (40 %) that had a mammogram within the previous 2 years was lower than the national average (73.2 %) for African American women [11], Majority of the study participants rated their health status as good or very good, and most had visited a primary care provider within the previous 2 years. It was not clear how these women pay for the health care services since 41 % did not have any form of health care coverage. The percent of uninsured in the study population was over three times higher than the 3-year (2010–2013) average for people without insurance in Kansas (12.9 %), Missouri (14.0 %), and nationally for all races (15.8 %) and for Blacks (19.8 %) [12].

**Table 2:** Health care access (N = 29)

Question	N	Percent
*How would you rate your health?*	*29*	
Very good	11	37.9
Good	14	48.3
Fair	4	13.8
*Where do you get most of your health care?*	29	
Community health clinic	2	6.9
Primary care doctor	18	62.1
Free clinic	2	6.9
Health department	6	20.7
Other	1	3.4
*When was your last routine check up?*	*29*	
Less than a year	11	39.3
1–2 years	10	35.7
2–5 years	1	3.6
5 or more years	2	7.1
Don’t know/not sure	3	10.7
Never	1	3.6
*How long since last teeth cleaning?*	*28*	
Less than a year	10	35.7
1–2 years	6	21.4
2–5 years	3	10.7
5 or more years	1	3.6
Never	8	28.6
*Do you currently have health insurance?*	*26*	
No insurance	11	42.3
Private insurance	9	34.6
Other	4	15.4
Prefer not to respond	2	7.7
*Have you ever had a clinical breast exam?*	*26*	
Yes	19	73.1
*How long since your last clinical breast exam?*	*25*	
Less than a year	7	28.0
1–2 years	6	24.0
2–5 years	2	8.0
5 or more years	3	12.0
Never	4	16.0
Not sure/don’t know	3	12.0
*Have you ever had a mammogram?*	*22*	
Yes	10	45.4
*How long since your last mammogram?*	*10*	
Less than a year	3	30.0
1–2 years	1	10.0
2–5 years	2	20.0
5 or more years	4	40.0
*Have you ever had a Pap test?*	*27*	
Yes	20	74.1
*How long since your last Pap test?*	*25*	
Less than a year	11	40.7
1–2 years	5	18.5
2–5 years	2	7.4
5 or more years	2	7.4
Never	5	18.5
Not sure/don’t know	2	7.4

Italicized texts represent total number of respondents per question

The high percent (41 %) of uninsured in the study population was surprising because 70 % was employed, suggesting that some of the women may be under-employed or working for establishments or businesses that do not offer health care coverage, or they may not consider health insurance important as most countries in Africa run their health systems with payer fee. One study limitation is that immigration status was not requested. That information may have explained the reason for the high percent uninsured in this population as immigration status has been shown to influence access to health insurance and health care services [8]. The American Cancer Society reported that nationally, 68 % of uninsured women in the United States did not get mammograms within the past 2 years [13]; thus, lack of health insurance is a huge barrier to regular breast cancer screening.

### Breast cancer knowledge, risk perception, and assessment

Because of increasing rates of non-communicable diseases in African countries and in African-born individuals in the United States, some sections of our survey focused on perceptions of cancer risk, and assessment of risk of breast and cervical cancer in our study population. Only 20 % of participants agreed that they were not at risk of breast cancer. Most (92.9 %) participants agreed that breast cancer can be treated if detected early, 72.4 % strongly disagreed that breast cancer is a curse from God, and 53.5 % either strongly agreed or agreed that breast cancer can disappear following prayer. A preliminary assessment of participants’ cancer risks is summarized in Table 3. While Black African-born women, like black United States-born women, have a lower incidence of breast cancer than White women, the overall mortality from breast cancer in Black women is still highest among all racial/ethnic groups [10]. However, it is difficult to separate the contribution of African immigrants to the overall incidence reported for Blacks; peradventure there are some risk factors peculiar to this subgroup. Breast health programs that address prevention, diagnosis and treatment of breast cancer are scarce or non-existent in most countries in Sub-Saharan Africa [6]. Therefore, prior to their immigration to the United States, many Black African-born women would have very little knowledge of or exposure to breast health programs and standard breast cancer screening tests. This potential lack of breast cancer awareness among Black African-born women may increase the risk of late breast cancer diagnosis in this population.

**Table 3:** Breast cancer risk

Question	N	Percent
*Do you smoke?*	*26*	
No	26	100
*At what age did you have your first child?*	*14*	
19–24 years	7	50
25–30 years	7	50
*Have you been told by doctor or provider that you have breast cancer?*	*27*	
No	27	100
*Have anyone in your family, on your mother’s side or father’s side ever had breast cancer?*	27	
No	22	81.5
Yes	3	11.1
Don’t know/not sure	2	7.4
*Have you ever had a breast biopsy?*	*27*	
No	24	88.9
*Have you been told by a doctor or health professional that you have dense breast tissue?*	*28*	
No	26	92.8
Yes	1	3.6
Don’t know/not sure	1	3.6
*Have you ever had radiation therapy to the chest or neck area as treatment for another cancer?*	*28*	
No	27	96.4
Don’t know/not sure	1	3.6
*Including you, has anyone in your family ever been told or tested for hereditary breast cancer with changes in BRCA1/BRCA2 genes?*	*28*	
No	22	78.6
Yes	1	3.6
Don’t know/not sure	5	17.8

Italicized texts represent total number of respondents per question

### Social and health concerns

Participants identified high blood pressure, obesity, oral health, HIV and diabetes as the most common health problems in their African community, similar to health issues among African American women [10]. Lack of jobs, health care cost, language barrier, discrimination, and child care were the top five social concerns in the African community. Black United States-born women also face challenges with racial discrimination, jobs and health care costs, but not the added burden of language barrier experienced by many Black African-born women—a barrier that limits their understanding of health issues, and their ability to adequately communicate with health care providers and navigate the health care system.

## Conclusions

While the sample size for this study was small and participants were from a Midwestern African-born population, these preliminary results provide a snapshot of the health care access and needs of the larger population of African-born women in the United States because similar issues have been reported for African immigrants in various other regions of the United States [1, 6, 10]. Future studies are needed to examine in more detail the health issues in African-born individuals in the United States, identify socio-cultural characteristics that influence behavior and health, and develop interventions that are culturally tailored to this culturally heterogeneous group of Black people.
